# Evaluation of recombinant human IGF-1/IGFBP-3 on intraventricular hemorrhage prevention and survival in the preterm rabbit pup model

**DOI:** 10.1038/s41598-023-46611-0

**Published:** 2023-11-13

**Authors:** Claes Ekström, Niklas Ortenlöf, Amanda Kristiansson, Bo Holmqvist, Åsa Jungner, Suvi Vallius, Xiaoyang Wang, Ann Hellström, Norman Barton, Galen Carey, David Ley, Magnus Gram

**Affiliations:** 1 Pediatrics, Department of Clinical Sciences Lund, Lund University, Skåne University Hospital, Lund, Sweden; 2 Pediatrics, Department of Clinical Sciences Lund, Lund University, Lund, Sweden; 3 ImaGene-iT AB, Lund, Sweden; 4 Institute of Neuroscience and Physiology, Sahlgrenska Academy, Department of Obstetrics and Gynecology, University of Gothenburg, Gothenburg, Sweden; 5 Department of Clinical Neuroscience, Sahlgrenska Academy, University of Gothenburg, Gothenburg, Sweden; 6 Scientific Advisory Board, Oak Hill Bio Ltd, WA14 2DT, UK; 7 Takeda, Cambridge, MA USA

**Keywords:** Paediatric research, Neonatal brain damage, Pharmaceutics

## Abstract

Insulin-like growth factor-1 (IGF-1) is essential for normal brain development and regulates processes of vascular maturation. The pathogenesis of intraventricular hemorrhage (IVH) relates to the fragility of the immature capillaries in the germinal matrix, and its inability to resist fluctuations in cerebral blood flow. In this work, using different experimental setups, we aimed to (i) establish an optimal time-point for glycerol-induction of IVH in relation to time-point of recombinant human (rh) IGF-1/rhIGFBP-3 administration, and (ii) to evaluate the effects of a physiologic replacement dose of rhIGF-1/rhIGFBP-3 on prevention of IVH and survival in the preterm rabbit pup. The presence of IVH was evaluated using high-frequency ultrasound and post-mortem examinations. In the first part of the study, the highest incidence of IVH (> 60%), occurred when glycerol was administered at the earliest timepoint, e.g., 6 h after birth. At later time-points (18 and 24 h) the incidence decreased substantially. In the second part of the study, the incidence of IVH and mortality rate following rhIGF-1/rhIGFBP-3 administration was not statistically different compared to vehicle treated animals. To evaluate the importance of maintaining intrauterine serum levels of IGF-1 following preterm birth, as reported in human interventional studies, additional studies are needed to further characterize and establish the potential of rhIGF-1/rhIGFBP-3 in reducing the prevalence of IVH and improving survival in the preterm rabbit pup.

## Introduction

Every third infant born at extremely preterm (EPT) gestational age develops a cerebral hemorrhage, which frequently progresses to a severe lesion^1, 2^. To a high degree, these lesions develop into post-hemorrhagic conditions with subsequent impact on neurocognitive development^3^. Preterm cerebral intraventricular hemorrhage (IVH) occurs during the first 72 h of postnatal life, and subsequently the risk of developing a hemorrhage is vastly reduced^4, 5^. Despite significant overall progress in the neonatal care of EPT infants, there is currently no existing treatment that can prevent or decrease the risk of IVH, and there is a persisting overall hemorrhage (all grades) incidence of around 35% with grade 3 and 4 corresponding to about 10% in Sweden and 16% globally^6–8^.

The current knowledge on the pathogenesis of IVH comprises a multifactorial etiology that relates to the fragility of the capillary network in the germinal matrix and its inability to resist fluctuations in cerebral blood flow due to impaired cerebral autoregulation in the premature physiology^9^. Insulin-like growth factor-1 (IGF-1) is an important regulator of preterm brain development and is linked to several cell processes involving proliferation, apoptosis and as a mediator of neuronal growth^10^. This trophic factor has an essential role in early vascular maturation and promoting functional stability of vascular components^11^. Treatment with IGF-1, directed to enhance the maturation of structural vascular components, has been shown to improve vascular integrity in murine models of neo-vascularization and increase angiogenic processes^11, 12^. Human interventional studies have reported beneficial effects of increasing postnatal IGF-1 levels to intrauterine levels, including improved growth and reduced morbidities^10, 13–15^. Furthermore, a recent phase 2 randomized controlled trial presented promising results in reducing the occurrence of severe IVH in a very preterm population following continuous administration of recombinant human (rh) IGF-1/rhIGF binding protein (BP) 3 at a physiologic replacement dose compared to neonatal standard of care^16^.

In the preclinical study by Gram et al.^17^, the IGF-1 system was characterized in the preterm rabbit pup, an animal model that mimics both the overall physiological effects of organ prematurity in humans, including lungs and brain, as well as rapidly decreasing levels of IGF-1 after birth^10, 17, 18^. A pharmacokinetic evaluation of rhIGF-1/rhIGFBP-3 (1.0–8.0 mg/kg) concluded that administration of 8 mg/kg resulted in peak IGF-1 serum levels that corresponded to endogenous physiologic levels at birth, i.e. a replacement dose to mirror in utero levels, without any signs of toxicity/negative side-effects. It was further observed that administration of the protein complex rhIGF-1/rhIGFBP-3 was associated with an induction of angiogenesis- and extracellular matrix (ECM) related components, including angiopoietin-1, IGF-1, fibronectin-1, collagen type I alpha 1/procollagen-1, versican, and thrombospondin-1, in the choroid plexus 24 h post-injection. Based on these observations, it was suggested that treatment with IGF-1 early after birth could decrease vascular fragility and vulnerability to fluctuations in cerebral blood flow, thereby reducing the risk of vessel rupture and induction of IVH^17^.

The preterm rabbit pup model of glycerol-induced IVH was initially described by Conner et al.^19^, and has since been used repeatedly to study preterm IVH/germinal matrix hemorrhage (GMH) of the preterm infant^20–23^. Preterm rabbit pups respond to the glycerol-induced hyperosmolarity with many traits similar to those considered to be central in the development of the hemorrhage in human preterm infants^21^, including perturbations of cerebral blood flow that exerts mechanical stress on the structural integrity of the vessels, leading to a subsequent rupture and bleeding into the ventricles^20, 23^. When Conner et al. initially described the model, IVH was induced 24 h after birth in pups delivered on gestational day 28^19^. More commonly today, induction occurs within the first 2–3 h after birth in animals delivered on gestational day 29^20–23^. Importantly, Ballabh et al. showed that induction of IVH at 48 h postnatal age resulted in mild to moderate, but not severe, IVH^24^, thus suggesting that a later timepoint of induction might be feasible.

Based on the findings in the phase 2 trial^16^, i.e*.* indication of reduced occurrence of severe IVH in a very preterm population following administration of rhIGF-1/rhIGFBP-3, in combination with the angiogenesis-related effects of rhIGF-1/rhIGFBP-3 described by Gram et al.^17^ we investigated if IGF-1 might have an impact on the development of IVH in the preterm rabbit pup model of glycerol-induced IVH. In the first part, we aimed to establish an optimal time-point for glycerol-induction of IVH that would enable a sufficient time window for rhIGF-1/rhIGFBP-3 to improve vessel maturity. In the second part of the study, we investigated the potential preventive effect of exogenously administered IGF-1 on the incidence of IVH, when administered in a complex with IGFBP-3 (rhIGF-1/rhIGFBP-3) at a physiologic replacement dose of 8 mg/kg, as established in Gram et al.^17^ to mirror in utero levels.

## Results

### Rate of IVH induction

No spontaneous IVH was detected in experiment A or B prior to injection of glycerol. In experiment A, the incidence of IVH in preterm rabbit pups after intraperitoneal (i.p.) administration of a 50% glycerol solution was the highest when administered at 6 h postnatal age reaching an incidence of 66% (n = 4 out of 6) (Table 1). At later time-points of induction (18 and 24 h) the incidence decreased substantially (33%, n = 2 out of 6 in the 18 h group; and 14%, n = 1 out of 7 in the 24 h group) (Table 1). No animals in the group receiving glycerol at 12 h of age developed IVH in this experiment (0%, n = 0 out of 5) (Table 1). One (1) animal from each of the 6-, 12-, and 18-h groups, died prior to scheduled termination. No postmortem examination was undertaken on the animals in experiment A, and the animals were not included in the subsequent analysis.

**Table 1:** Incidence of IVH in experiment A.

Time-point of glycerol administration and group size (n)	IVH incidence at 24 h n/n and %	
6 h (7*)	4/6	66%
12 h (6*)	0/5	0%
18 h (7*)	2/6	33%
24 h (7)	1/7	14%

**Figure 1:**
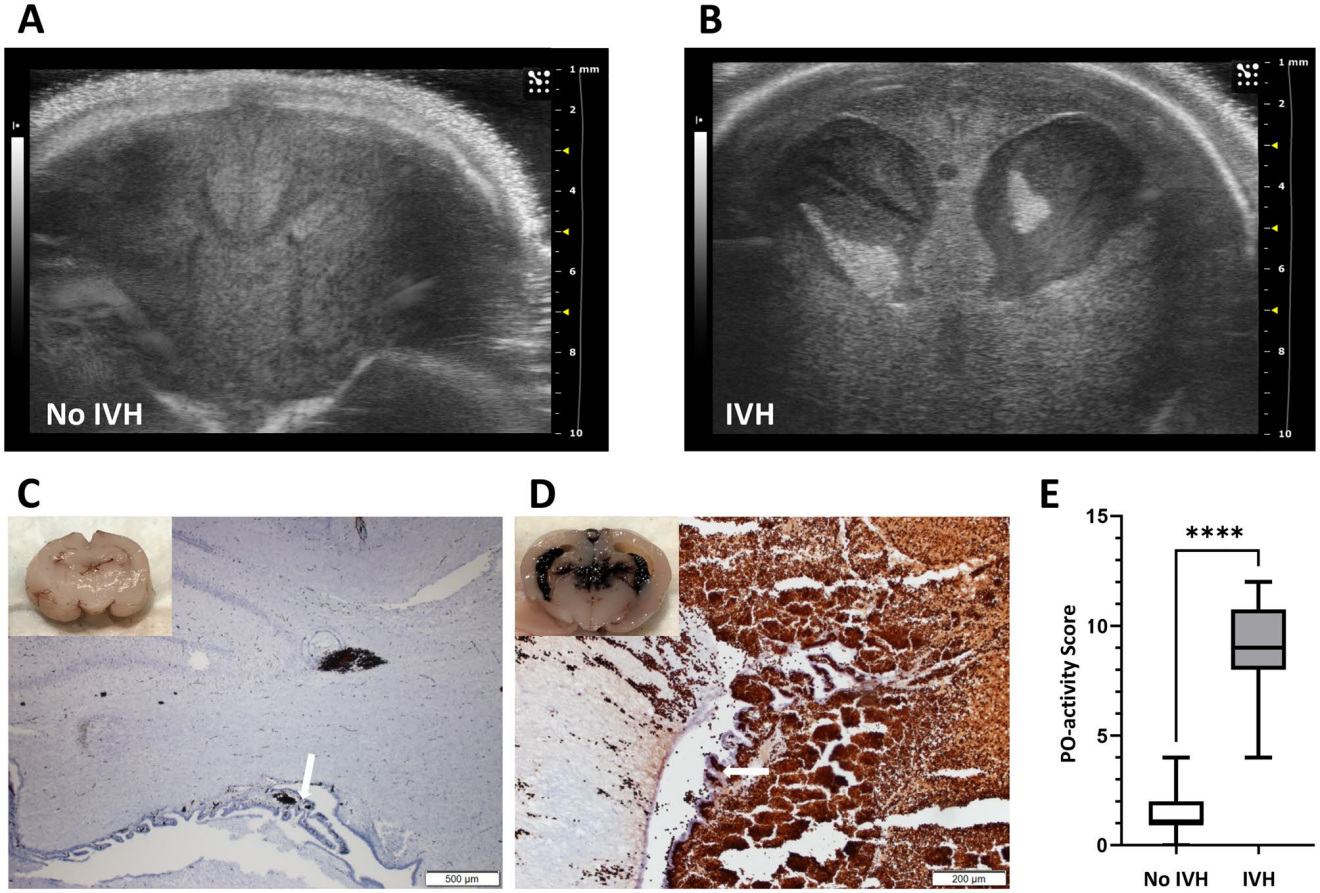
Evaluation of IVH-induction. Preterm rabbit pups without signs of spontaneous cerebral hemorrhage (as determined by HFU), were administered a single i.p. bolus injection of 50% (v/v) glycerol solution as described in experiment A and B (see “Methods” section for further details) to induce IVH. The extent of bleeding was assessed by HFU and histological analysis (as described in the “Methods” section) and bleedings were scored as IVH or No IVH. (**A**,**B**) Representative HFU images of a premature rabbit pup with No IVH (**A**) or with IVH (**B**). Ultrasound imaging was performed at 12-, 24- and 48 h post-glycerol administration. (**C**–**E**) Histological analysis of brains from experiment B. (**C**) Representative image of PO-activity labeling of a midbrain cryosection of a No IVH animal. White arrow indicate choroid plexus and adjacent ventricular space. Scalebar shows 500 μm. Upper left corner shows a macroscopic histomorphological image of a midsection of the brain from the same animal. (**D**) Representative image of PO-activity labeling of a midbrain cryosection of an IVH animal. White arrow indicate choroid plexus. Scalebar shows 200 μm. Upper left corner shows a macroscopic histomorphological image of a midsection of the brain from the same animal. (**E**) Total PO-activity scoring, evaluating the distribution and extent of the bleeding, in animals with No IVH (n = 58) or severe IVH (n = 19). For details of the experiment and results, including scoring of IVH and No IVH, see the “Methods” and “Results” sections. Results in (**E**) are presented as a boxplot with min and max values. Differences between groups were analyzed using a Mann–Whitney test. ****P < 0.0001.

Preterm rabbit pups without signs of spontaneous cerebral hemorrhage (as determined by high-frequency ultrasound, HFU) were administered a single i.p. bolus injection of 50% (v/v) glycerol solution at 6, 12, 18, or 24 h postnatal age to induce IVH. HFU assessed the extent of bleeding at 24 h post-glycerol administration and bleedings were scored as IVH or No IVH (see Fig. 1A,B for further details). *One animal of the group died before scheduled termination; no postmortem examination was undertaken, and the animal was not included in the subsequent analysis. For details of the experiment and results, see the “Methods” and “Results” sections, as well as Supplementary Table S1.

Based on these results, a glycerol administration time-point of 6 h postnatal age was selected as the time-point for induction of IVH in experiment B. Group sizes were set to n ≥ 33/experimental group to obtain a power of 0.8 (see statistical determinants in the “Methods” section).

### Incidence of IVH, sex distribution, and body weight

In total, 77 animals were included in experiment B, 38 in the group receiving rhIGF-1/rhIGFBP-3, and 39 in the vehicle administered group. Out of the 77 animals, 18 developed an IVH as determined by high-frequency ultrasound (HFU) at 24 (30) hours and 19 at 48 (54) hours post-glycerol administration, postnatal age indicated within brackets, corresponding to an IVH incidence of 23.4% and 24.7%, respectively.

No statistically significant difference in the incidence of IVH was observed between groups. The occurrence of IVH in the rhIGF-1/rhIGFBP-3 and vehicle administered control group at 48 h was 21% (n = 8 out of 38) and 28.2% (n = 11 out of 39; P-value 0.598), respectively (Table 2).

**Table 2:** Incidence of IVH in experiment B.

Treatment groups	Number of animals	IVH at 24 h n (%) and P-value		IVH at 48 h n (%) and P-value	
rhIGF-1/rhIGFBP-3	38	8 (21.0)	0.788	8 (21.0)	0.598
Vehicle	39	10 (25.6)		11 (28.2)	

Preterm rabbit pups were injected s.c. with rhIGF-1/rhIGFBP-3 (8 mg/kg/dose) or vehicle at approximately 3 h of postnatal age and thereafter every 12 h at an additional 4 consecutive time-points. A single i.p. bolus of 50% glycerol solution was administered at 6 h of postnatal age to induce IVH. The extent of bleeding was assessed by HFU at 24 and 48 h post-glycerol administration and bleedings were scored as IVH or No IVH (see Fig. 1A,B for further details). P-values from Fischer’s exact test comparing rhIGF-1/rhIGFBP-3 vs. Vehicle at 24 and 48 h post-glycerol administration, respectively. For details of the experiment and results see “Methods” and “Results” sections, as well as Supplementary Table S2.

Distribution of sex was similar in the rhIGF-1/rhIGFBP-3 group (17 male, 21 female) and the vehicle group (17 male, 22 female), with no significant difference (P-value 0.999). Distribution of sex in the treatment groups was not different (P-value 0.180) when comparing animals with IVH, where the rhIGF-1/rhIGFBP-3 group consisted of 5 male and 3 female pups and the vehicle administered group consisted of 3 male and 8 female pups.

During the 48 h of the study, all animals displayed a body weight gain with no statistically significant difference between groups, with a total weight gain of 6.4 ± 3.7 g (mean ± SD) for the vehicle administered group and 4.9 ± 3.3 g for the rhIGF-1/rhIGFBP-3 group, respectively.

### Histological analysis confirmed ultrasound evaluations

Brain tissue was evaluated by histological analysis, as described in the “Methods” section, to validate the accuracy of the ultrasound examinations and to identify presence of any additional bleedings within the parenchyma. Macroscopic histomorphological evaluation displayed no bleedings in animals scored as No IVH in the ultrasound examination, exemplified by Fig. 1C. All animals scored as IVH in the ultrasound examination were confirmed as IVH in the histological examination, as exemplified in Fig. 1D. Peroxidase (PO) activity staining further confirmed the results of ultrasound and histological examinations (Fig. 1E).

### Treatment with rhIGF-1/rhIGFBP-3 and rate of survival

**Figure 2:**
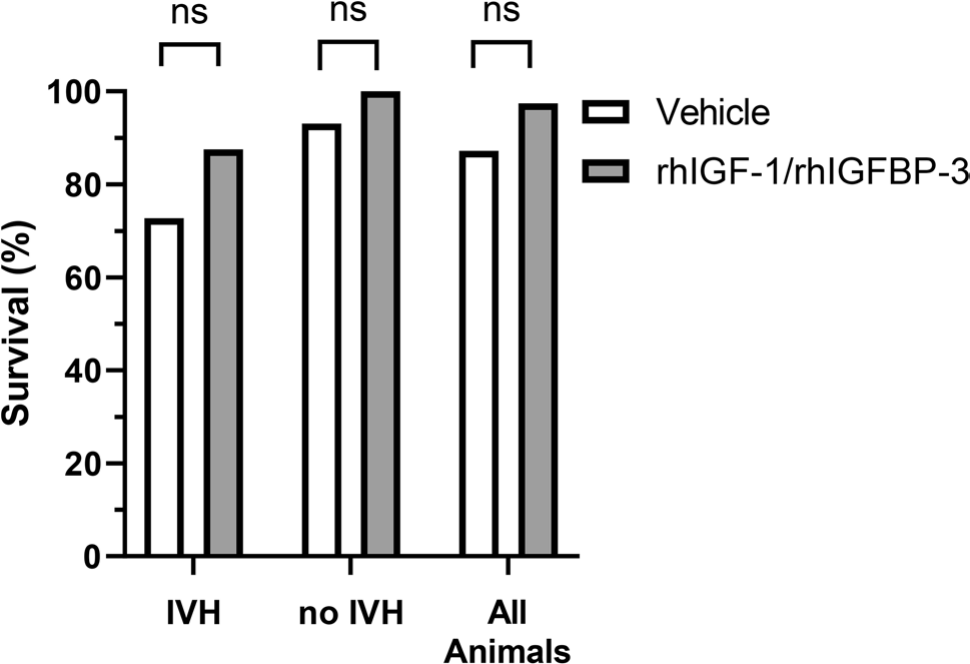
Survival in experiment B. Preterm rabbit pups were s.c. administered with rhIGF-1/rhIGFBP-3 (8 mg/kg/dose, n = 38) or vehicle (n = 39) at approximately 3 h of age, and thereafter every 12 h for a total of 5 administrations. A single i.p. bolus of 50% glycerol solution was administered at 6 h of age to induce IVH. Animals were followed up until 54 h of postnatal age, corresponding to 48 h post-glycerol administration, at which point the extent of bleeding was assessed by HFU (bleedings were scored as IVH or No IVH), and survival was evaluated. For details of the experiment and results see “Methods” and “Results” sections. Results are presented in a bar graph. Differences between groups were analyzed using Fischer’s exact test with P-value and the difference between proportions as attributable risk in percent (%).

The survival at 48 h following IVH was 72.7% (n = 8 out of 11) in the vehicle administered group and 87.5% (n = 7 out of 8) in the rhIGF-1/rhIGFBP-3 administered group (Fig. 2 and Table 3). In animals with No IVH, survival of vehicle administered animals was 92.8% (n = 26 out of 28) compared to 100% (n = 30 out of 30) in the rhIGF-1/rhIGFBP-3 administered animals (Fig. 2 and Table 3). The overall survival, i.e., regardless of developing an IVH or not, was 87.2% (n = 34 out of 39) in the vehicle administered animals as compared to 97.4% (n = 37 out of 38) in the rhIGF-1/rhIGFBP-3 administered animals (Fig. 2 and Table 3).

**Table 3:** Mortality in experiment B.

Treatment group (n)	Mortality at 48 h					
	IVH-animals n/n (%) and P-value		No IVH-animals n/n (%) and P-value		All animals n/n (%) and P-value	
rhIGF-1/rhIGFBP-3 (38)	1/8 (12.5)	0.602	0/30 (0)	0.228	1/38 (2.6)	0.200
Vehicle (39)	3/11 (27.3)		2/28 (7.1)		5/39 (12.8)	

Preterm rabbit pups were injected s.c. with rhIGF-1/rhIGFBP-3 (8 mg/kg/dose) or vehicle at approximately 3 h of postnatal age and thereafter every 12 h at an additional 4 consecutive time-points. A single i.p. bolus of 50% glycerol solution was administered at 6 h of postnatal age to induce IVH. The mortality was continuously evaluated until 54 h of postnatal age, corresponding to 48 h post-glycerol administration. P-values from Fischer’s exact test comparing rhIGF-1/rhIGFBP-3 vs. Vehicle in the IVH, No IVH, and all animals, respectively. For details of the experiment and results, see “Methods” and “Results” sections.

### Serum levels of IGF-1

**Figure 3:**
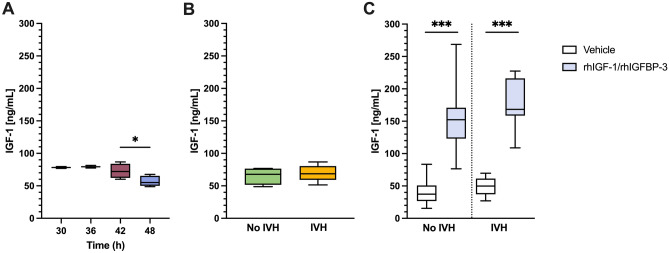
Serum IGF-1. (**A**,**B**) Preterm rabbit pups without signs of spontaneous cerebral hemorrhage (as determined by HFU), were administered a single i.p. bolus injection of 50% (v/v) glycerol solution at 6-, 12-, 18- or 24 h postnatal age to induce IVH. The extent of bleeding was assessed by HFU at 24 h post-glycerol administration and bleedings were scored as IVH or No IVH. (**A**) Endogenous serum IGF-1 protein level was determined in preterm rabbit pups at 30–48 h postnatal age (n = 2–5), as described in the “Methods” section. (**B**) Endogenous serum IGF-1 protein level was determined in pups with IVH (n = 9) or No IVH (n = 4) 24 h post-glycerol administration, as described in the “Methods” section. (**C**) Preterm rabbit pups were injected with rhIGF-1/rhIGFBP-3 s.c. (8 mg/kg/dose) or vehicle at approximately 3 h of age, and thereafter every 12 h at an additional 4 consecutive time-points. A single i.p. bolus of 50% glycerol solution was administered at 6 h of age to induce IVH. The extent of bleeding was assessed by HFU at 48 h post-glycerol administration, corresponding to 54 h postnatal age, and bleedings were scored as IVH or No IVH. Serum levels of IGF-1 were determined in pups with IVH (rhIGF-1/rhIGFBP-3 group, n = 8; vehicle administered group, n = 11) or No IVH (rhIGF-1/rhIGFBP-3 group, n = 30; vehicle administered group, n = 28), as described in the “Methods” section. Results are presented as a boxplot with min and max values. Differences between groups were analyzed using unpaired *t*-test [between 42- and 48 h in (**A**)]. *P < 0.05, ***P < 0.001. For details of the experiment and results, see “Methods” and “Results” sections.

Analysis of circulating levels of endogenous IGF-1 at termination time-points in experiment A displayed a decline from 78 ng/ml (n = 2) at 30 h postnatal age to 57 ng/ml (n = 5) at 48 h postnatal age, with a significant decrease between 42 (73 ng/ml, n = 4) and 48 h (Fig. 3A). Endogenous IGF-1 serum levels were not statistically different in animals that developed IVH (70 ± 12 ng/ml, n = 9) from No IVH animals (65 ± 13 ng/ml, n = 4; P-value 0.539) at 48 h (Fig. 3B). These results correspond well with previously published data 48 h after birth^17^.

In experiment B, serum levels of IGF-1 were significantly increased at 48 (54) hours following rhIGF-1/rhIGFBP-3 administration in both No IVH animals (149 ± 42 ng/ml; n = 29) and in IVH animals (179 ± 41 ng/ml; n = 7) as compared to vehicle administered animals (No IVH, 39 ± 16 ng/ml, n = 27; IVH, 48 ± 15 ng/ml, n = 7; P-value < 0.001) (Fig. 3C). Serum levels of IGF-1 were not statistically different in animals that developed an IVH compared to No IVH animals (P-value 0.18) following administration of rhIGF-1/rhIGFBP-3 (Fig. 3C).

## Discussion

Low serum levels of IGF-1 after premature birth have been associated with increased rates of bronchopulmonary dysplasia (BPD), retinopathy of prematurity (ROP), decreased brain growth, and impaired neurodevelopmental outcome^25, 26^. In accordance with these observations, in a recent clinical trial, a trend towards a decreased rate of severe IVH was observed in EPT infants following supplementary treatment with rhIGF-1/rhIGFBP-3^16^. In the current study, we investigated if raising the serum IGF-1 levels to intrauterine concentrations, by repeated administration of 8 mg/kg/dose of rhIGF-1/rhIGFBP-3, would have a preventive effect on the development of glycerol-induced IVH in the preterm rabbit pup. With the experimental outline used in this study, we did not observe any significant decrease in the incidence of IVH, after administration of rhIGF-1/rhIGFBP-3 as compared to vehicle administered animals.

The brain of the preterm rabbit pup delivered three days prior to term (i.e. E29) corresponds approximately to human gestational week (GW) 24–27. During this gestational period, the vascularization of the germinal matrix is at its peak and subsequently decreases until GW 35–36^27–30^. Neovascularization in the germinal matrix and subventricular zone makes the sprouting vessels susceptible to fluctuations in cerebral blood flow as the nascent vessels, that constitutes the blood brain barrier, lack covering pericytes, have a deficiency of fibronectin in the basal lamina, and the protective end feet of the glia-cells are deficient in glial fibrillary acid protein^22, 31^. The choroid plexus, which constitutes the blood cerebrospinal fluid barrier, is comprised of an epithelium, fenestrated vessels and a stromal compartment. During the period of EPT birth, the choroid plexus is still in development and immature^32, 33^. Despite differences between these vascular compartments’ morphogenesis and development, they are both inherently vulnerable to perturbations in cerebral blood flow in these areas, that is most pronounced during early postnatal life, and thus constitutes a high risk of IVH development. Of note, we have previously described the choroid plexus to be an essential site for vessel rupture in the preterm rabbit pup^21, 47^. In the preterm infants with IVH, most bleedings occur during the first 72 h after birth, and subsequently the risk of developing a bleeding is vastly reduced^4, 5^. In previous studies, glycerol administration in the established preterm rabbit pup IVH model has most often been administered at approx. 2–3 h after birth^20–23^. However, since Gram et al. showed that the effects of administered IGF-1 were the largest after 24 h in terms of formation, maturation and regulation of cerebrovascular structure^17^, the aim in this study was to postpone the administration of glycerol as much as possible, thus increasing the time span for IGF-1 to affect IVH incidence. As mentioned above, a later induction of IVH has previously been described by both Conner et al., who initially described the model, with IVH induction 24 h after birth^19^, and Ballabh et al., who induced IVH at 48 h postnatal age. Hence, in experiment A, glycerol was administered 6-, 12-, 18- and 24 h after birth. In line with clinical observations and previous studies in the preterm rabbit pup model of glycerol-induced IVH^4, 5, 24^, we observed that IVH was induced at the highest rate when glycerol was administered early, i.e. at 6 h after birth. The lower rates at later time-points (18- and 24 h after birth) suggest that essential maturational and protective mechanisms are operational, rendering the physiology less vulnerable to vascular perturbations. Using a later time-point of IVH-induction would have been preferred, however, based on the results of experiment A this would have resulted in a significantly lower rate of IVH, and hence a significantly larger number of animals would have been required. We concluded that this would neither be feasible nor ethically acceptable.

Prospective Swedish national cohort studies have shown that while the survival of EPT infants born before 28 GWs has increased, the incidence of severe IVH (grade 3–4) has not decreased^8, 34^. Thus, the need for preventive and/or therapeutic intervention remains high. In line with the report of a potential protective effect of rhGF-1/rhIGFBP-3 on the development of both mild and severe IVH, there is a growing body of evidence that replacement therapy with rhIGF-1/rhIGFBP-3 may have beneficial effects on the underlying mechanisms involved in the development of germinal matrix IVH^16, 35^. For instance, Gram et al. reported that systemic administration of rhIGF-1/rhIGFBP-3 in preterm rabbit pups caused an upregulated gene expression and increase of the corresponding protein levels of factors controlling vessel maturation and structure in the choroid plexus^16, 17^. Similarly, Christiansen et al. showed that treatment with rhIGF-1/rhIGFBP-3 promoted cerebellar protein synthesis rates and brain maturation in preterm piglets^36, 37^. However, a recent study by Sharma et al. showed that blockage of the IGF-1 receptor at a later time point, postnatal day 23, in the preterm rabbit pup resulted in an improvement in cognitive deficits^38^. On the other hand, Trofinetide, a small synthetic analog tripeptide derivative of IGF-1, was recently approved in the United States for the treatment of a rare childhood neurodevelopmental disorder, Rett syndrome, where administration is associated with significant symptom improvements^39^. Together this further indicates that IGF-1 treatment might have an early, and time-dependent window for optimal therapeutic effect in treatment of IVH.

In addition to a decreased risk of developing IVH, it has also been reported that infants supplemented to higher levels of circulating IGF-1 have a significant reduction in risk of developing severe BPD compared to standard neonatal care^16^. These findings are further supported by preclinical research, showing that postnatal supplementary treatment with IGF-1 has maturational effects on several organ systems in neonatal animal models. Treatment with rhIGF-1/rhIGFBP-3 was established to promote lung angiogenesis and restored lung function in a rat model of BPD^40^. Furthermore, in a preterm piglet model of necrotizing enterocolitis (NEC), Holgersen et al. showed that continuous infusion of rhIGF-1/rhIGFBP-3 resulted in trophic effects on intestinal growth and increased protein synthesis potentially coupled to an overall increased viability compared to control animals^41^. These findings may well support that physiologic supplementation of circulating IGF-1 levels promotes maturation of structural components vital for vascular integrity that protects against fluctuations in cerebral blood flow, and that maintaining intrauterine serum levels of IGF-1 during the first fragile days after EPT birth could be key for reducing the prevalence of IVH and increasing the survival^17, 22, 42^. Nonetheless, in our study we did not observe any statistically significant effect on occurrence of IVH or effect on survival compared to vehicle administered animals following subcutaneous (s.c.) administration of rhIGF-1/rhIGFBP-3, 8.0 mg/kg/dose twice daily. Importantly though, based on the rate of IVH induction observed in experiment A (> 60%), a group size estimation for experiment B was established. However, the rate of IVH obtained in experiment B (~ 25%) was significantly lower, thus clearly influencing the ability to evaluate the impact on both IVH-induction and mortality. Consequently, additional studies are needed to further characterize and establish the role of IGF-1 in preterm cerebral IVH.

Although the preterm rabbit pup model of glycerol-induced IVH provides a robust model mimicking the development of IVH, it has limitations. As described above, we experienced a large variability in the incidence of induced IVH between experiments A and B, with significantly lower IVH incidence in experiment B (24% as compared to 66% in experiment A) constituting definite consequences for the group size estimations. Although there are no apparent differences between the study setups, e.g., brand and dose of glycerol, breeding, husbandry and feeding regimens of pups and rabbits, the difference in IVH induction between experiment A and B can be due to other factors that are less controllable, such as seasonal variability between the litters, skewed genetic background of bucks and/or does, or other coincidences or factors not considered.

Cerebral ultrasound is a non-invasive, bedside neuroimaging tool for detection and follow up of IVH in the premature infant and is widely implemented in clinical neonatal care^43^. In our preclinical model of glycerol-induced cerebral hemorrhage, we have adopted a similar approach, examining the rabbit pups for cranial bleeding using HFU with a 40 MHz probe. This method has been described in detail previously^21^, showing that HFU enables precise evaluation of regional distribution of hemorrhage and is a sensitive, quantitative measure of ventricular size. In this study, we report that HFU provides a highly accurate tool for diagnostic evaluation of IVH in the preterm rabbit pup with 100% agreement with histomorphological evaluation.

In this study, we administered rhIGF-1/rhIGFBP-3 twice daily at 8 mg/kg/dose, aiming to achieve circulating concentration of IGF-1 similar to those obtained in preterm infants with intravenous (i.v.) doses of 250–400 μg/kg/24 h. The dosing regimen was chosen based on the pharmacokinetics-pharmacodynamics described in Gram et al., where the maximum dose administered (8 mg/kg), gave peak concentrations similar to concentrations reported in utero and displayed significant effects on vascular maturation without toxicity. However, in contrast to the clinical study, the dosing regimen of 8 mg/kg/dose twice daily does not achieve a stable circulatory concentration, but displays levels decreasing below target levels between dosing time-points. Based on this, continuous infusion could have been a more adequate approach. However, continuous infusion is difficult to achieve and maintain in preterm rabbits due to the immature vessels and that restraining the animals for a prolonged period would be unethical. To circumvent this, new administration regimens should be explored. For example, a dosing scheme with mixed administration routes e.g., i.v. combined with repeated i.p. and/or s.c. administrations could be considered. Thus, it could be speculated that obtaining a higher area under the curve and/or a steady state of circulatory IGF-1 levels could have a more pronounced preventive effect on the development of IVH^17, 22^.

In conclusion, here we did not observe a decrease in the incidence of IVH following administration of rhIGF-1/rhIGFBP-3 twice daily from birth. Importantly though, we believe that the data presented here in combination with previously reported clinical data warrants further animal studies with larger animal cohorts and/or different administration regimens to investigate and establish the potential of supplementation of rhIGF-1/rhIGFBP-3 in EPT infants.

## Methods

### Animals

Animal studies were conducted in accordance with Swedish legislation and approved by the Swedish Animal Ethics Committee in Malmö-Lund, Sweden. Reporting follows the ARRIVE guidelines^44^. The rabbit model for preterm birth with glycerol-induced IVH was used in accordance with previous description^17, 21^, containing some adjustments as described below in section “Experimental setup”. A half-breed between New Zealand White and Lop was used (Löberöd, Sweden).

The experiments were performed on a total of 139 rabbit pups from 19 litters delivered preterm via caesarean section (c.s.) after the does were anesthetized with i.v. propofol (5 mg/kg, Primen Pharmaceuticals Oy, Helsinki, Finland) on embryonic day 29 (full term = 31–32 days). After delivery, highly experienced animal laboratory staff handled, fed, and nursed the pups. The pups were dried and placed in an infant incubator set at 30 °C and 60% humidity.

### Experimental setup

**Figure 4:**
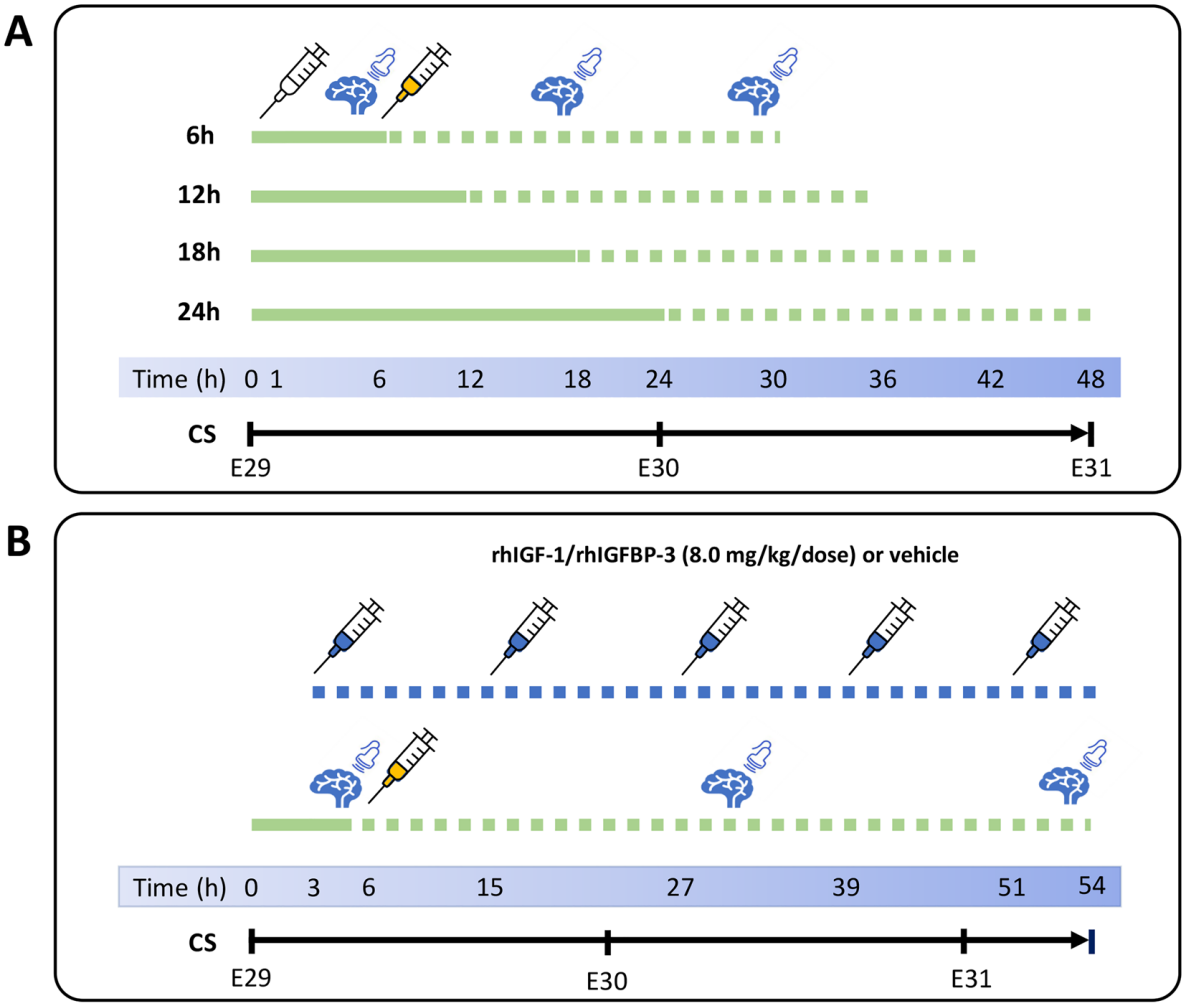
Overview of experimental setup of experiment A and B. The details of the experiments are described in the “Methods” section. Below is a brief summary of the details and a description of the abbreviations and symbols included in the figure. (**A**) Experiment A. Preterm rabbit pups were delivered by cesarean section (CS) at a gestational age of 29 days (E29) and placed in an infant incubator. All animals were injected s.c. with saline (white syringe) after birth, and subsequently allocated to experimental groups (6 h, 12 h, 18 h or 24 h, n = 6–7/group). Animals were administered a single i.p. bolus injection of 50% (v/v) glycerol solution at 6-, 12-, 18- or 24 h after birth in order to induce IVH. The continuous green line represents the time of resp. i.p. administration of glycerol (yellow syringe), whereas the dotted green line represents follow up time post-glycerol administration. The extent of bleeding was assessed by HFU at 12- and 24 h post-glycerol administration and bleedings were scored as IVH or No IVH (see Fig. 1A,B for further details). Animals were euthanized 24 h post-glycerol administration and samples were collected as described in the “Methods” section. (**B**) Experiment B. Preterm rabbit pups were delivered by cesarean section (CS) at a gestational age of 29 days (E29) and placed in an infant incubator. Animals were s.c. injected with 8 mg/kg/dose rhIGF-1/rhIGFBP-3 or vehicle (blue syringes) at 3 h after birth and thereafter every 12 h at 4 consecutive time-points (for a total of 5 administrations). Animals were administered a single i.p. bolus injection of 50% (v/v) glycerol solution at 6 h of age in order to induce IVH (yellow syringe). The extent of bleeding was assessed by HFU at 24- and 48 h post-glycerol administration and bleedings were scored as IVH or No IVH (see Fig. 1A,B for further details). Animals were euthanized at 48 h post-glycerol administration and samples were collected as described in the “Methods” section. *E* embryonic day.

Following preterm birth, animals were marked and randomized to experimental groups, treatment regimens, and termination time-points with equal distribution based on their body weight and litter. The experimental setup of experiments A and B are shown in detail in Fig. 4.

#### Investigation on the IVH-induction window

In experiment A, the animals were injected s.c. after birth with a single bolus (100 μl) of sterile isotonic saline (0.9% NaCl, B Braun, Melsungen, Germany) to mimic the injections in experiment B. At approximately 5 h after birth, animals were monitored with HFU (Vevo 2100, VisualSonics Inc., Toronto, ON, Canada) with an MS-550D 40 MHz transducer to scan for any spontaneous cranial bleedings as described previously^21^. All animals without signs of cerebral hemorrhage received a single bolus of 50% (v/v) sterile glycerol solution (6.5 g/kg, Teknova, Hollister, US) given as an i.p. injection at 6, 12, 18, or 24 h after birth, in accordance with the allocation, after randomizing pups from nine does as described above, to respective experimental group. Two consecutive ultrasound scans were performed at 12 and 24 h post-glycerol administration. Animals were terminated 24 h post-glycerol administration, corresponding to a postnatal age of 30 (6 h glycerol administration group), 36 (12 h glycerol administration group), 42 (18 h glycerol administration group) or 48 (24 h glycerol administration group) hours, respectively.

#### Preventive effect of rhIGF-1/rhIGFBP-3 on glycerol-induced IVH

In experiment B, the animals, after randomizing pups from ten does as described above, were injected with rhIGF-1/rhIGFBP-3 s.c. at 8 mg/kg/dose (supplied and prepared in vehicle solution as described by Takeda Pharmaceutical Company Ltd, Boston, MA, USA), or saline vehicle (0.9% NaCl, B Braun), at approximately 3 h of age, and thereafter every 12 h at four consecutive time-points, for a total of 5 administrations. A single i.p. bolus of 50% glycerol solution (6.5 g/kg, Teknova) was administered at 6 h of age, i.e., 3 h after the first rhIGF-1/rhIGFBP-3 administration. HFU scans were performed as described in experiment A every 24 h post-glycerol administration to monitor the development and severity of IVH. Pups with severe IVH according to the ultrasound scan, were assigned to the IVH group (see Fig. 1B for a representative image), and those without detectable IVH at all time-points were assigned to the control group (see Fig. 1A for a representative image). Reproducibility and accuracy of ventricular measurements in this animal model using HFU have been described previously^21^. The extent of bleeding was scored according to a simplified version of the standardized scale from Volpe^45^. All examinations were performed with the operator blinded to the treatment group. Animals were terminated 48 h post-glycerol administration, corresponding to a postnatal age of 54 h. Animals that were euthanized due to poor physical health status, or died prior to the scheduled termination, were subject to post-mortem ultrasound examinations and allocated to the IVH or No IVH group.

### Tissue collection and processing

Animals were euthanized by decapitation at respective endpoint. Blood was collected in Li-Heparin and serum tubes (Microvette, Sarstedt, Germany) and centrifuged according to the instructions from the manufacturer. Plasma and serum were immediately frozen and stored at − 80 °C until further analysis. An ear biopsy used for sex determination was collected, snap-frozen and stored at − 80 °C until further analysis.

Following termination, brains were dissected out from the skull and immersion fixated for 24 h in freshly prepared 4% paraformaldehyde solution (PFA, in 0.1 M phosphate buffer, pH 7.4), with a change to fresh PFA after 3–6 h, at 4 °C. Brains were then cryoprotected by sequential immersion in 15% sucrose (diluted in phosphate buffer saline, PBS, 0.1 M, pH 7.4) for 6 h, followed by 25% sucrose (diluted in PBS) for another 6 h. Brains were mounted in TissueTec (Sakura Finetek, Torrance, CA, USA) and frozen (at around − 60 °C) in cryomolds, on dry ice in isopentane (2-methylbutane, Sigma-Aldrich, St. Louis, MO, USA). Sections (12 µm) were cut on a cryotome (Microm, HM 500OM, Microm Laborgeraete GmbH, Walldorf, Germany) and collected on SuperFrost plus slides (Menzel, Braunschweig, Germany). The brains were cryosectioned by coronal dissection in three blocks (forebrain, midbrain, and hindbrain) and stored at − 20 °C.

### Hematoxylin–eosin

Sections were stained with hematoxylin–eosin (HE) to enable macroscopic neuroanatomical evaluation. Sections were air-dried, at room temperature (RT) or 37 °C, for 20–30 min, rinsed in PBS, 2 × 5 min, followed by a rinse in H_2_O for 1 min. Sections were immersed in Mayer’s Hematoxylin (Histolab, Gothenburg, Sweden) for 2 min, followed by 3 × 1 min rinses in H_2_O. Sections were then immersed in sodium bicarbonate (0.1%) for 1 min followed by immersion in H_2_O for 2 × 1 min. Sections were immersed in 70% ethanol for 2 min, followed by Eosin (Histolab, 0.2% diluted in 70% ethanol acidified with glacial acetic acid) for 3 min. Sections were dehydrated in alcohol, 96% × 2, and 100% × 2 for 3 min in each solution, and in xylene, 100% for > 10 min. Sections were mounted in Pertex (Histolab) and cover slipped.

### Peroxidase histochemistry

We followed an adapted protocol of the enhanced peroxidase reaction of cryosections to detect PO activity, as a means of evaluating the distribution of the bleeding^46, 47^. Briefly, sections were air-dried, at RT or 37 °C, for 20–30 min, rinsed in PBS 2 × 10 min. The PO-reaction was performed in a solution containing 3,3′-diaminobenzidine (DAB, 0.5 mg/ml diluted in PBS, Sigma-Adrich) containing 0.015% H_2_O_2_ (Merck, Darmstadt, Germany), for 10 min at RT. Sections were then rinsed in PBS 3 × 5 min, and in H_2_O for 1 min. Counterstaining was performed with hematoxylin (HTX, Mayer’s, Histolab), via immersion of sections in HTX for 2 min, and rinses in H_2_O for 3 × 1 min. Sections were dehydrated in alcohol, 70% for 1 min, 96% for 2 × 5 min, and 100% for 2 × 7 min, and in xylene, 100% for 2 × 5 min. Sections were mounted in Pertex (Histolab) and cover slipped.

### Histological evaluation

Histological evaluation of the degree of IVH was undertaken to compare and validate the HFU examination. The evaluations were made in three steps using both non-stained and tissue stained with HE and PO-activity (as described above).

First, an overall image analysis was performed including a macroscopic morphological assessment for the locality of any bleeding. Second, the brain was visually evaluated for the extent of any bleeding located in the arachnoidea, parenchyma, ventricle, cerebellum or brainstem. Third, an overall scoring of the bleeding was performed for evaluation of the degree of ventricular dilatation, findings of any tissue atrophy or necrosis in the three coronally sectioned blocks. When conducting the image analysis and scoring, the evaluator was blinded to treatment group.

### Serum IGF-1 levels

The serum concentration of IGF-1 was determined using a human IGF-1 ELISA kit (Mediagnost, Reutlingen, Germany). The analysis was performed according to the manufacturer’s instructions.

### Sex determinations

Determinations of rabbit sex was done by confirming the presence of the sex-determining region Y gene (gene ID: 100328958) in the rabbit genome using PCR and gel electrophoresis visualization as described before^17^. Briefly, DNA was extracted using DNeasy Blood and Tissue Kit (Qiagen, Hilden, Germany) according to the manufacturer’s instructions. 1 μl of DNA (range: 100–200 ng/μl) was used in respective PCR reaction (30 cycles at 57 °C) with the following primers: Sense: TGCAATACAGGAGGAACACG, Antisense: AGCAAACTGTCGCTCTTCTG. Presence of a band at approx. 299 bp determined the animal as male, and no corresponding band determined the animal as female.

### Group size estimations and data analysis

A group size estimation, based on the prevalence of IVH in experiment A and a proposed treatment effect of 50%, based on data derived from the clinical study reported by Ley et al.^16^, was made to calculate the anticipated number of animals in respective groups for experiment B. Calculations were made with the following estimations, a power of (1 − β = 0.80) and Type I error of (α = 0.05), generating the group sizes described under section "Preventive effect of rhIGF-1/rhIGFBP-3 on glycerol-induced IVH".

Statistical significance was calculated using a Mann–Whitney test or using Student’s *t*-test assuming parametrical data. Categorical outcome analysis was done on contingency data with Fischer’s exact test with P-value and the difference between proportions as attributable risk in percent (%). Statistical tests are specified in the figure legends. When possible, animals that died or were euthanized before scheduled termination were included in the statistical analysis. P-values < 0.05 were considered significant. Statistical analyses were performed using GraphPad Prism (GraphPad Prism 9; GraphPad, San Diego, CA, USA).

### Supplementary Information

Supplementary Tables.

## Data Availability

Data available upon request from the corresponding author.
