# Author Correction: Evaluation of recombinant human IGF-1/IGFBP-3 on intraventricular hemorrhage prevention and survival in the preterm rabbit pup model

**DOI:** 10.1038/s41598-024-60175-7

**Published:** 2024-04-24

**Authors:** Claes Ekström, Niklas Ortenlöf, Amanda Kristiansson, Bo Holmqvist, Åsa Jungner, Suvi Vallius, Xiaoyang Wang, Ann Hellström, Norman Barton, Galen Carey, David Ley, Magnus Gram

**Affiliations:** 1 Pediatrics, Department of Clinical Sciences Lund, Lund University, Skåne University Hospital, Lund, Sweden; 2 Pediatrics, Department of Clinical Sciences Lund, Lund University, Lund, Sweden; 3 ImaGene-iT AB, Lund, Sweden; 4 Institute of Neuroscience and Physiology, Sahlgrenska Academy, Department of Obstetrics and Gynecology, University of Gothenburg, Gothenburg, Sweden; 5 Department of Clinical Neuroscience, Sahlgrenska Academy, University of Gothenburg, Gothenburg, Sweden; 6 Scientific Advisory Board, Oak Hill Bio Ltd, WA14 2DT, UK; 7 Takeda, Cambridge, MA USA

Correction to: *Scientific Reports* 10.1038/s41598-023-46611-0, published online 13 November 2023

The original version of this Article contained an error in the Results section, under the subheading ‘Treatment with rhIGF-1/rhIGFBP-3 and rate of survival’,

“The survival at 48 h following IVH was 63.6% (n = 7 out of 11) in the vehicle administered group and 87.5% (n = 7 out of 8) in the rhIGF-1/rhIGFBP-3 administered group (Fig. 2 and Table 3).”

now reads:

“The survival at 48 h following IVH was 72.7% (n = 8 out of 11) in the vehicle administered group and 87.5% (n = 7 out of 8) in the rhIGF-1/rhIGFBP-3 administered group (Fig. 2 and Table 3).”

Additionally, as the result of this error, Figure 2 was incorrect. The original Figure 2 and accompanying legend appear below.

**Figure 2:**
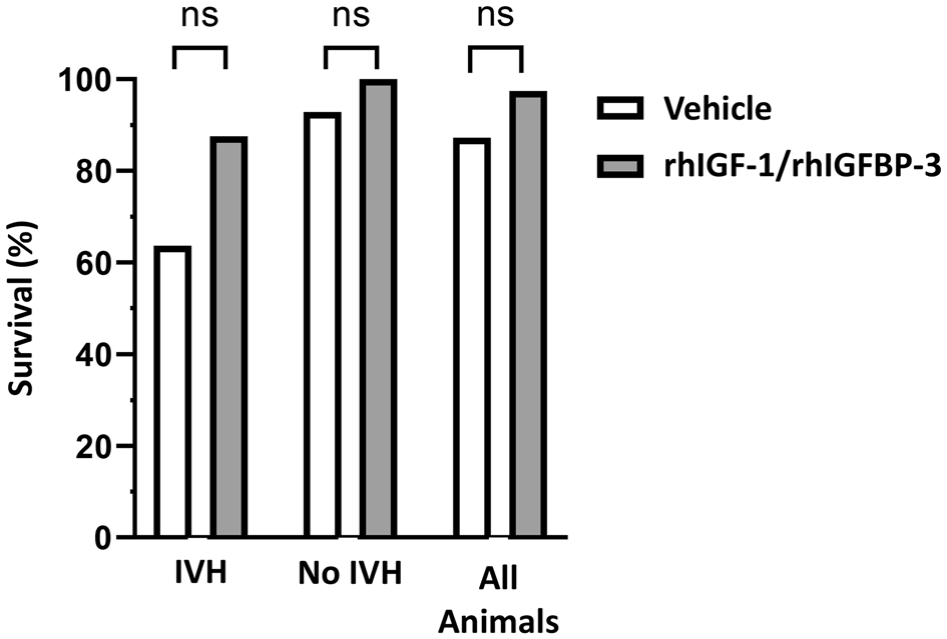
Survival in experiment B. Preterm rabbit pups were s.c. administered with rhIGF-1/rhIGFBP-3 (8 mg/kg/dose, n = 38) or vehicle (n = 39) at approximately 3 h of age, and thereafter every 12 h for a total of 5 administrations. A single i.p. bolus of 50% glycerol solution was administered at 6 h of age to induce IVH. Animals were followed up until 54 h of postnatal age, corresponding to 48 h post-glycerol administration, at which point the extent of bleeding was assessed by HFU (bleedings were scored as IVH or No IVH), and survival was evaluated. For details of the experiment and results see “Methods” and “Results” sections. Results are presented in a bar graph. Differences between groups were analyzed using Fischer’s exact test with P-value and the difference between proportions as attributable risk in percent (%).

The original Article has been corrected.

