# Supplementary material for: Evaluation of recombinant human IGF-1/IGFBP-3 on intraventricular hemorrhage prevention and survival in the preterm rabbit pup model
Source: Sci Rep. 2023 Nov 13;13:19847. doi: 10.1038/s41598-023-46611-0 (PMC10645867; doi:10.1038/s41598-023-46611-0)
Supplement: Supplementary file 1 — Supplementary Tables. [file 41598_2023_46611_MOESM1_ESM.docx]

**Supplementary Information**

**Supplementary Table S1. Incidence of IVH by litter in experiment A**

|  | IVH incidence at 24 hours | | | | | | | | | | | | | | | | | |
| --- | --- | --- | --- | --- | --- | --- | --- | --- | --- | --- | --- | --- | --- | --- | --- | --- | --- | --- |
| **Time-point of glycerol admin. (n/)** | **Litter 1** | | **Litter 2** | | **Litter 3** | | **Litter 4** | | **Litter 5** | | **Litter 6** | | **Litter 7** | | **Litter 8** | | **Litter 9** | |
|  | **n/n** | **%** | **n/n** | **%** | **n/n** | **%** | **n/n** | **%** | **n/n** | **%** | **n/n** | **%** | **n/n** | **%** | **n/n** | **%** | **n/n** | **%** |
| 6 hours (6) | - | - | - | - | - | - | 0/1 | 0 | 3/4 | 75 | - | - | - | - | 1/1 | 100 | - | - |
| 12 hours (5) | 0/2 | 0 | - | - | - | - | 0/1 | 0 | 0/1 | 0 | 0/1 | 0 | - | - | - | 0 | - | - |
| 18 hours (6) | - | - | 1/1 | 100 | - | - | 0/2 | 0 | - | - | 0/1 | 0 | 0/1 | 0 | - | 0 | 1/1 | 100 |
| 24 hours (7) | - | - | 0/2 | 0 | 1/1 | 100 | 0/2 | 0 | 0/1 | 0 | - | - | - | - | - | 0 | 0/1 | 0 |

Preterm rabbit pups without signs of spontaneous cerebral hemorrhage (as determined by HFU) were administered a single i.p. bolus injection of 50% (v/v) glycerol solution at 6, 12, 18, or 24 hours postnatal age to induce IVH. HFU assessed the extent of bleeding at 24 hours post-glycerol administration and bleedings were scored as IVH or No IVH (see Main article; Figure 2A-B for further details). For details of the experiment and results, see the Methods and Results sections.

**Supplementary Table S2. Incidence of IVH by litter in experiment B**

| **Treatment groups** | **Litter** | **Number of animals n (%)** | **IVH at 24 hours** | | **IVH at 48 hours** | |
| --- | --- | --- | --- | --- | --- | --- |
|  |  |  | **n/n** | **%** | **n/n** | **%** |
| rhIGF-1/rhIGFBP-3 | 1 | 4 (44) | 0/4 | 0 | 0/5 | 0 |
| Vehicle |  | 5 (56) | 1/5 | 20 | 2/5 | 40 |
| rhIGF-1/rhIGFBP-3 | 2 | 4 (50) | 1/4 | 25 | 1/4 | 25 |
| Vehicle |  | 4 (50) | 1/4 | 25 | 1/4 | 25 |
| rhIGF-1/rhIGFBP-3 | 3 | 4 (44) | 2/4 | 50 | 2/4 | 50 |
| Vehicle |  | 5 (56) | 2/5 | 40 | 2/5 | 40 |
| rhIGF-1/rhIGFBP-3 | 4 | 5 (46) | 0/6 | 0 | 0/6 | 0 |
| Vehicle |  | 6 (54) | 1/5 | 20 | 1/5 | 20 |
| rhIGF-1/rhIGFBP-3 | 5 | 4 (50) | 0/4 | 0 | 0/4 | 0 |
| Vehicle |  | 4 (50) | 0/4 | 0 | 0/4 | 0 |
| rhIGF-1/rhIGFBP-3 | 6 | 3 (43) | 1/3 | 33 | 1/3 | 33 |
| Vehicle |  | 4 (57) | 1/4 | 25 | 1/4 | 25 |
| rhIGF-1/rhIGFBP-3 | 7 | 6 (55) | 2/6 | 33 | 2/6 | 33 |
| Vehicle |  | 5 (45) | 1/5 | 20 | 1/5 | 20 |
| rhIGF-1/rhIGFBP-3 | 8 | 4 (44) | 1/4 | 25 | 1/4 | 25 |
| Vehicle |  | 5 (56) | 1/5 | 20 | 1/5 | 20 |
| rhIGF-1/rhIGFBP-3 | 9 | 1 (100) | 0/1 | 0 | 0/1 | 0 |
| Vehicle |  | - | - | - | - | - |
| rhIGF-1/rhIGFBP-3 | 10 | 2 (50) | 1/2 | 50 | 1/2 | 50 |
| Vehicle |  | 2 (50) | 2/2 | 100 | 2/2 | 100 |

Preterm rabbit pups were s.c. injected with rhIGF-1/rhIGFBP-3 (8 mg/kg/dose) or vehicle at approximately 3 hours of postnatal age and thereafter every 12 hours at an additional 4 consecutive time-points. A single i.p. bolus of 50% glycerol solution was administered at 6 hours of postnatal age to induce IVH. The extent of bleeding was assessed by HFU at 24 and 48 hours post-glycerol administration and bleedings were scored as IVH or No IVH (see Figure 2A-B in Main article for further details). For details of the experiment and results see Methods and Results sections.
